# Experimental Measurement of Diffusion Coefficient of Polyimide Film for Capacitive Humidity Sensors

**DOI:** 10.3390/polym14224910

**Published:** 2022-11-14

**Authors:** Jianyun Wu, Wenhe Zhou, Xiaowei Wang, Shicheng Li

**Affiliations:** 1 School of Environmental and Municipal Engineering, Lanzhou Jiaotong University, Lanzhou 730070, China; 2 Gansu Institute of Architectural Design and Research CO., Ltd., Lanzhou 730030, China

**Keywords:** humidity sensor, polyimide film, humidify-sensitive properties, effective diffusion coefficient

## Abstract

Polyimide (PI) film is widely used as the key component of the capacitive humidity sensor, whose diffusion coefficient has a significant impact on the sensor’s dynamic characteristics, but is rarely discussed. This paper provides a test method and processes for effective diffusion coefficients of water molecules in self-synthesis PI films. The films were formed by four ingredients (PMDA-ODA, BPDA-ODA and BPDA-BAPP, PMDA-BAPP) with PI acid concentrations of 23%, 20%, 17% and 15%, and tested in temperatures of 20 °C, 35 °C and 50 °C, respectively. The results indicated that BPDA-BAPP film was good as a moisture sensitive film, whose average effective diffusion coefficient was 2.709 × 10^−14^ m^2^/s. The temperature of the environment had a significant effect on the humidity-sensitive properties, but the PI acid concentration effect could be indirect.

## 1. Introduction

Humidity sensors are broadly applied in diverse disciplines and areas of human activity, such as environmental and meteorological monitoring; material preparation; agricultural, food processing and quality monitoring; pharmaceutical preparation; wearable and flexible equipment; health services; high energy physics applications; as well as heating, ventilation, and air-conditioning [1,2]. Among all types of humidity sensors, polymer capacitive sensors have been demonstrated to have better stability, particularly in high humidity levels [2]. Although the characteristics of capacitive humidity sensors are mainly credited to their moisture sensitive films [3,4,5,6], most of the research on sensor improvement has been focused on the sensors, instead of the films.

The transfer processes of water molecules in the porous membrane are dependent on many factors, such as diffusion coefficient, pore performance, relative humidity, etc., and are usually accompanied by the condensation of water molecules and chemical reactions. Although researchers have continuously tried to interpret it, the transfer mechanism of water molecules in such films has still not been clarified. Among all types of humidity-sensing films, such as naphthalene diimide, small organic molecules are gaining more attention. However, their performance needs deeper investigation and verification before marketization. Due to their more mature application and outstanding properties, such as high sensitivity, low manufacturing cost, ease and diversity of fabrication methods, and compatibility with flexible substrates, the polymers have been widely chosen as humidity sensing film in humidity sensors [7,8,9,10,11,12,13]. The performance parameters of the polymers correlated with the humidity sensors are scarcely found, which is a bottleneck to improving the characteristics of humidity sensors, especially dynamic characteristics. Several papers on the sorption and transport behavior of water vapor in PI films used for separation processes have been found [14,15], which is helpful for humidity sensor research.

This paper aims to show a practical test system and method by which the effective diffusion coefficients of porous film can easily be obtained. In addition, the humidity sensing properties of the four PI films mentioned in this paper will be analyzed, which will be helpful in predicting and optimizing the dynamic characteristics of the humidity sensor with PI film.

## 2. The Principle

### 2.1. Humidity Sensing Mechanism of the Sensor

Equations (1) and (2) indicate the working mechanism of the parallel plate capacitor [16,17]. In the equilibrium state, it is easy to obtain the sensor capacitance according to the volume share of water molecules in the film, *γ*.

(1) $$C=\varepsilon_{0}\varepsilon_{s}\frac{S}{l}$$

(2) $$\varepsilon_{s}={\left\{\gamma \left(\varepsilon_{w}^{1/3}-\varepsilon_{p}^{1/3}\right)+\varepsilon_{p}^{1/3}\right\}}^{3}$$

where *C* is the sensor capacitance, s and l are sensor area and distance between electrodes (film thickness), respectively, and *ε*_0_, *ε*_p_, *ε*_w_ and *ε*_s_ are the dielectric constants of vacuum, dry film, water and moisture film, respectively.

In the transient condition, it is necessary to obtain mole concentration of water moisture in the film, N, before calculating its volume share, *γ*, and sensor capacitance, *C*. Equation (3) indicates the transfer processes of water molecules in the PI film. It is clear that the effective diffusion coefficient of water molecule in the film, *D*, is the key factor in these processes.

(3) $$\partial N\left(x,y,z,t\right)/\partial t=D[\partial^{2}\left(x,y,z,t\right)/\partial x^{2}+\partial^{2}\left(x,y,z,t\right)/\partial y^{2}+\partial^{2}\left(x,y,z,t\right)/\partial z^{2}]$$

where *x*, *y* and *z* are three coordinates, and *t* is the time.

### 2.2. The Test Principle

According to the standard [18] and steady state method [19], the relation between the mass flow rate of vapor and its partial pressure gradient is shown as Equation (4).

(4) $$g_{v}=-\delta \cdot \nabla p_{v}$$

where *g*_v_ is the mass flow rate of vapour, kg/(m^2^·s); *δ* is the permeability coefficient of vapour, kg/(m·s·Pa); and *p*_v_ is the partial pressure of vapour, Pa.

Under the isothermal condition, and, based on Fick’s first law, one-dimensional transfer of vapor and the linear distribution of vapor partial pressure and relative humidity in PI film, the the mass flow rate of vapour can be calculated as Equation (5).

(5) $$g_{v}=-\rho \cdot D\cdot \frac{\partial w}{\partial \varphi }\cdot \frac{1}{p_{\mathrm{vs}}}\cdot \frac{p_{v2}-p_{v1}}{l}$$

where *D* is the effective diffusion coefficient, m^2^/s; *ρ* is the performance density of PI film, kg/m^3^; *w* is the moisture mass percentage of PI film, kg/kg; *φ* is the relative environmental humidity at experimental temperature, % RH; and *p*_vs_ is the saturated vapor pressure at experimental temperature, Pa.

According to Equations (4) and (5), the effective diffusion coefficient *D* can be written as Equation (6) [20]. As long as *δ* and *w*(*φ*) are obtained, the effective diffusion coefficient *D* can be calculated.

(6) $$D=\frac{\delta \cdot p_{\mathrm{vs}}}{\rho }\cdot \frac{1}{\partial w/\partial \varphi }$$

According to [18], *δ* can be expressed as Equation (7).

(7) $$\delta =\frac{\Delta m}{A\cdot \Delta t}\cdot \frac{l}{2}\cdot \frac{1}{p_{\mathrm{vs}}\cdot \Delta \varphi }$$

where Δ*m* is the mass change of PI films, kg; Δ*φ* is the environment humidity change of adsorption process, %RH; Δ*t* is the diffusion time, s; and *A* is the area of PI film, m^2^.

Under the isothermal condition and a certain gas pressure, the absorbing capacity of gas on a solid surface is within a certain amount. Based on the Peleg model (8) [21] and the experiment data, the function curve between film mass change and relative humidity can be fitted. Additionally, the four coefficients in the Peleg model, a_1_, a_2_, b_1_ and b_2_, can be provided with the help of ‘Origin 2018′ software.

(8) $$w\left(\varphi \right)=\frac{m\left(\varphi \right)-m_{0}}{m_{0}}=a_{1}\varphi^{b_{1}}+a_{2}\varphi^{b_{2}}$$

where *m*_0_, *m*(*φ*) are film mass at dry state and wet condition, respectively, and a_1_, b_1_, a_2_, b_2_, are coefficients (b_1_ < 1, b_2_ > 1).

### 2.3. The Test System

**Figure 1:**
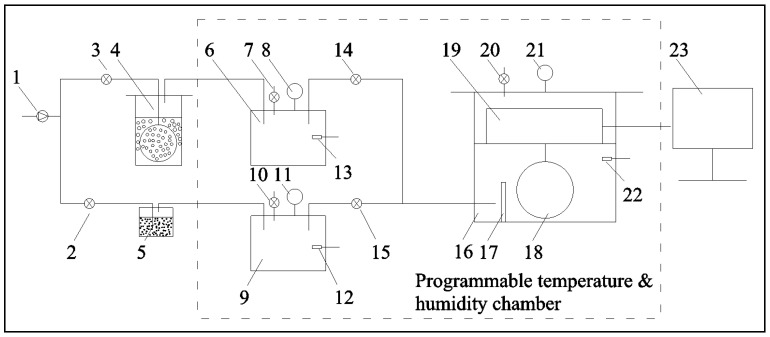
Schematic diagram of the test system.

Based on Equations (4)–(8), a test system was constructed and is shown as Figure 1, which mainly consisted of air pump 1, moisture air generator 4 and its tank 6, dry air generator 5 and its tank 9, absorption chamber 16, electronic scales 19, data recorder 23, some valves (2, 3, 7, 10, 14, 15 and 20), pressor gages (8, 11 and 21) and sensors (12, 13 and 22). The effect of baffle 17 was to slow down the air flow. The parts surrounded by dotted lines were kept in the isothermal chamber during the test. Table 1 lists information of the main equipment.

### 2.4. The Test Procedure

Before the test, PI film was hung on the hook of electronic scales 19 in absorption chamber 16 to obtain its mass, and air pump 1 pushed the air into generator 4 which was full of water, and generator 5 which was full of calcium chloride anhydrous, respectively. Then, the moister air (about 40% RH) and the dry air (about 20% RH) were stored in tanks 6 and 9, respectively.

The dry air from tank 9 entered absorption chamber 16 until the film reached mass balance. Then, the humidity air from tank 6 entered absorption chamber 16 until the equilibrium state was reached. During this process, the time, film mass and relative humidity were all recorded. Finally, the dry air entered absorption chamber 16 again, until the equilibrium state was reached once more. Then the test was repeated. Each test condition was repeated three times to obtain an average value.

Each permeability coefficient *δ*_i_ and moisture absorption *w*_i_ corresponding to relative humidity *φ*_i_ of each test condition was calculated successively, according to Equations (4) and (5). Then, the formulation of *w*(*φ*) and its coefficients a and b were obtained by fitting the curve of *w*_i_. Finally, the diffusivity of the film was obtained, based on Equation (6).

## 3. Preparation of Polyimide Films

### 3.1. Subsection

To obtain the PI humidity sensing films, PMDA (1,2,4,5-Benzenetetracarboxylic anhydride), BPDA (3,3’,4,4’-biphenyltetracarboxylic di-anhydride), BAPP (4,4’-(4,4’-Isopropylidenediphenyl-1,1’-diyldioxy)dianiline), ODA (Octadecylamine) and DMAC (N,N-Dimethylacetamide) were prepared, which are listed in Table 2.

**Figure 2:**
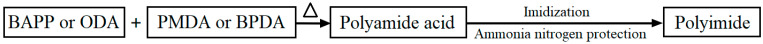
The flow chart of PI film preparation.

Figure 2 shows the synthesis process of the PI films. Firstly, binary anhydride and diamine with the same molar mass were put into DMAC, and stirred and heated to obtain PI acid. Different concentrations of PI acid were attained by changing the ratio of the total mass of binary anhydride and diamine, and the mass of DMAC. Then, the PI acid was dropped on a glass sheet gripped by a spin coater. Finally, the glass sheets were put into an electric furnace controlled by a program. The heat and imidization program were as follows: from room temperature to 100 °C for 40 min, 100 °C to 150 °C for 30 min, 150 °C to 200 °C for 30 min and 300 °C to 290 °C for an hour. According to the above method, four PI films were obtained, which were PO (PMDA-ODA), BO (BPDA-ODA), BB (BPDA-BAPP) and PB (PMDA-BAPP), with four concentrations of PI acid, respectively, of 23%, 20%, 17% and 15%.

### 3.2. Film Analysis

**Figure 3:**
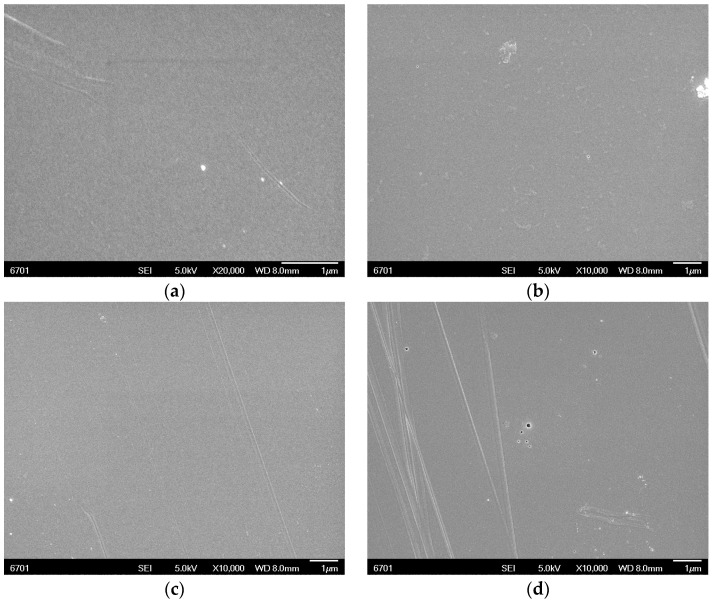
SEM photos of the four films: (**a**) PO film; (**b**) BO film; (**c**) BB film; (**d**) PB film.

With the help of the step profiler, the thicknesses of the PI film samples were obtained. Figure 3 shows SEM photos of the four films. The film surfaces were all smooth, which indicated that the dianhydride and diamine had dissolved and reacted well in DMAC.

**Figure 4:**
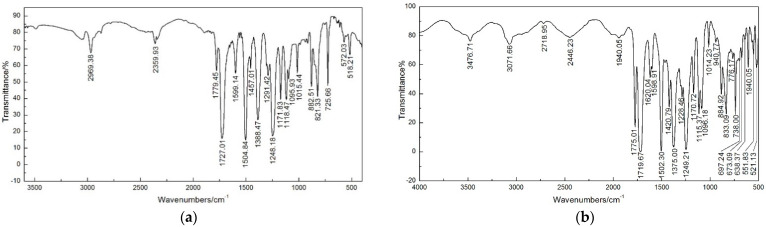

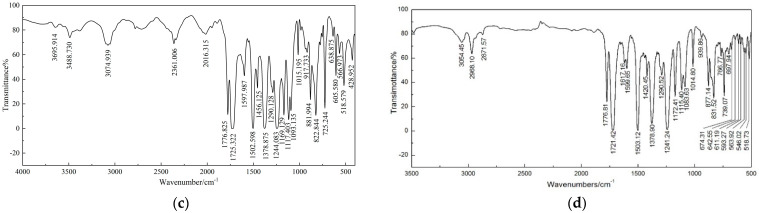
The FTIR spectra of the four PI films: (**a**) PB film; (**b**) BO film; (**c**) PO film; (**d**) BB film.

In addition, the four films were analyzed by Fourier transform infrared (FTIR) spectra, and recorded, as shown in Figure 4. Spectra in the optical range of 400 cm^−1^ to 4000 cm^−1^ were obtained by averaging 32 scans at a resolution of 4 cm^−1^. The characteristic peaks of resultant PI are assigned in Table 3. By comparing the infrared spectra of the four films, no characteristic absorption peaks of polyamide acid were found at 1660 cm^−1^, 2900 cm^−1^ and 3200 cm^−1^, which indicated that the polyamide acid had been fully imidized.

## 4. The Test Results and Analysis

### 4.1. The Reagent Effect

**Figure 5:**
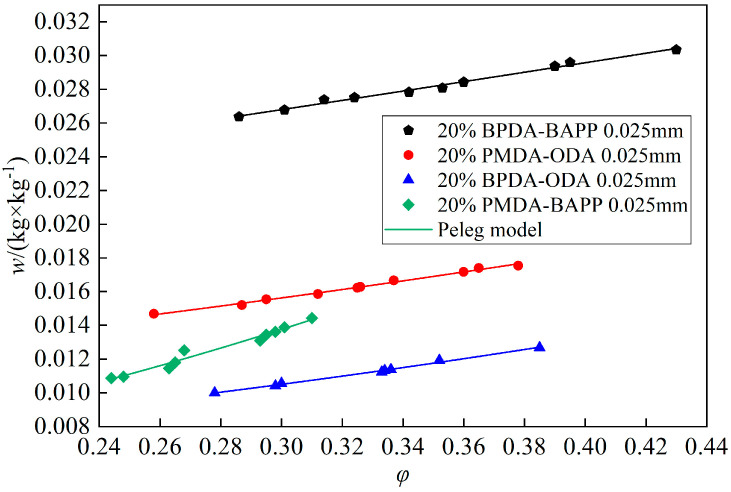
Absorption isothermal curves of the four different types of PI films at 20 °C.

According to above test method, the four PI films of PO, BO, BB and PB, with a same thickness of 0.025 m, area of 0.01 m^2^ and PI acid concentration of 20%, were tested at a temperature of 20 °C. Figure 5 shows the coupled absorption isothermal curves of the four different types of PI films based on the Peleg model [21]. The *w*(*φ*) curves of the four films all increased linearly with relative humidity. This could be due to the greater porosity of BB film resulting in the highest absorbing capacity of water molecules, and was found to be good as a moisture sensitive film, whose average value was 2.836 × 10^−2^ kg/kg in the test, followed by PO, BO and PB. The curved slope of the BO film was obviously larger than the other films, and it was also good as a moisture sensitive film because it was more sensitive to humidity change.

(9) $$w{\left(\varphi \right)}_{20\%\mathrm{BB}}^{20{}^{\circ}C}=0.01062\varphi^{4.6\times 10^{-15}}+0.02936\varphi^{1.46918}$$

(10) $$w{\left(\varphi \right)}_{20\%\mathrm{PO}}^{20{}^{\circ}C}=0.00428\varphi^{1.25\times 10^{-16}}+0.08286\varphi^{1.8}$$

(11) $$w{\left(\varphi \right)}_{20\%\mathrm{BO}}^{20{}^{\circ}C}=0.01937\varphi^{5.08\times 10^{-10}}+0.02809\varphi^{1.1051}\;$$

(12) $$w{\left(\varphi \right)}_{20\%\mathrm{PB}}^{20{}^{\circ}C}=0.00618\varphi^{1.386\times 10^{-16}}+0.03154\varphi^{1.6504}$$

**Figure 6:**
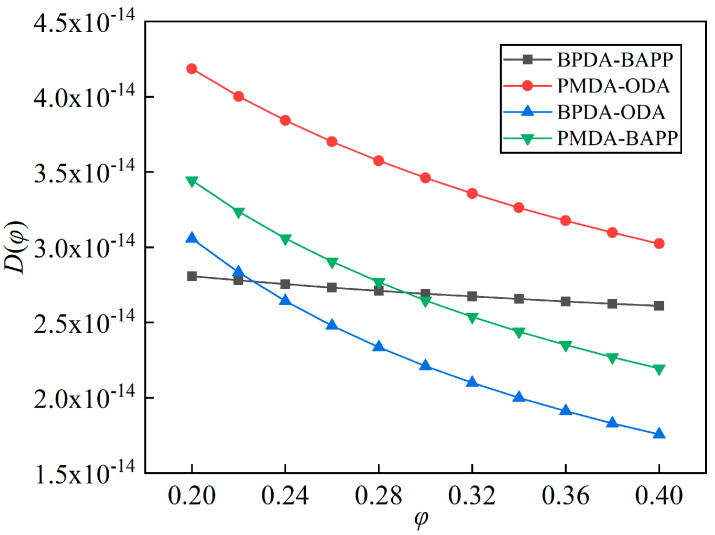
Effective diffusion coefficients of the four different types of PI films at 20 °C.

Equations (9)–(12) describe the coupled formulas with correlation coefficients of 0.9935, 0.9697, 0.9908 and 0.9947, respectively. The effective diffusion coefficients of the four films were calculated, and are shown in Figure 6. With the increase in relative humidity, more pores of the film were occupied, the diffusion pathways of water molecules in the film were narrower and the diffusion resistances were larger, so the effective diffusion coefficient decreased, which was a negative correlation with the absorption isothermal curve. PO film had the highest value of effective diffusion coefficient, whose average value was 3.605 × 10^−14^ m^2^/s in the range of 20% RH–40% RH, which could be caused by it containing a greater number of connected pores, than the other films. The average diffusivities of BB, BO and PB were 2.709 × 10^−14^ m^2^/s, 2.407 × 10^−14^ m^2^/s, and 2.82 × 10^−14^ m^2^/s, respectively, in the range of 20% RH–40% RH. BB film had a smoother curve, which could have been caused by its greater number of porous, and fewer number of hydrophilic groups. More porosity means more diffusion pathways, and fewer hydrophilic groups means less diffusion resistance; as result, the increase in relative humidity had lesser impact on the effective diffusion coefficient of water molecules in BB film.

### 4.2. The Concentration Effect

**Figure 7:**
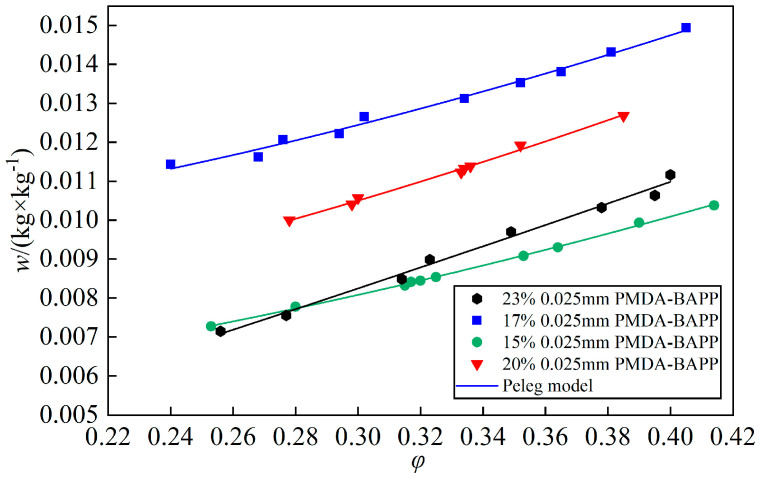
Absorption isothermal curves of PI films with different PI acid concentrations at 20 °C.

Four PI films with PI acid concentrations of 23%, 20%, 17% and 15% were prepared, respectively, which all had 0.01 m^2^ area and 0.025 m thickness. The test was conducted at 20 °C. Figure 7 shows the test results and their *w*(*φ*) curves coupled by the Peleg model. The *w*(*φ*) curves of the four films had a similar tendency of linear increase with relative humidity. The moisture absorption of PB film with 17% PI acid was the highest, which was 1.248 × 10^−2^ kg/kg in the test range, followed by 20%, 23% and 15% PI acids. This could have been caused by different porosities. It indicated that the moisture absorption of PI film depends on many factors, but not for PI acid concentration.

(13) $$w{\left(\varphi \right)}_{23\%\mathrm{PB}}^{20{}^{\circ}C}=0.00126\varphi^{1.21\times 10^{-16}}+0.02789\varphi^{1.14947}$$

(14) $$w{\left(\varphi \right)}_{17\%\mathrm{PB}}^{20{}^{\circ}C}=0.00902\varphi^{4.2\times 10^{-15}}+0.02941\varphi^{1.78553}\;$$

(15) $$w{\left(\varphi \right)}_{15\%\mathrm{PB}}^{20{}^{\circ}C}=0.005\varphi^{2.038\times 10^{-16}}+0.02522\varphi^{1.74545}$$

**Figure 8:**
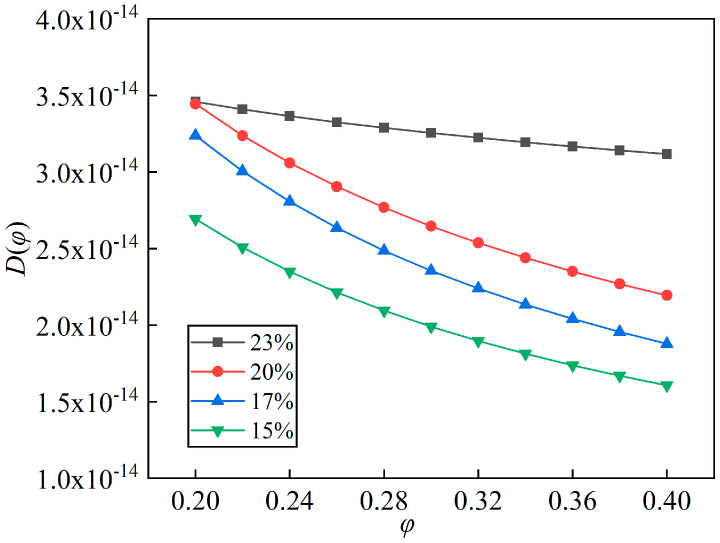
Effective diffusion coefficients of PI films with different PI acid concentrations at 20 °C.

Four coupled formulas are shown as Equations (9), (13)–(15), whose correlation coefficients were 0.9907, 0.9947, 0.9907, and 0.9988, respectively. According to the above method, the effective diffusion coefficients of water molecules in films were calculated, and the results are shown in Figure 8. The curves all displayed a declining trend with the increasing relative humidity, in contrast with the absorption isothermal curves. Although the porosity was not the largest, the PI film with 23% PI acid concentration had the highest diffusivity, whose average value was 3.288 × 10^−14^ m^2^/s in the range of 20% RH–40% RH, followed by the films with PI acid concentrations of 20% (2.82 × 10^−14^ m^2^/s), 17% (2.559 × 10^−14^ m^2^/s) and 15% (2.150 × 10^−14^ m^2^/s). This could be explained as follows: when the environmental relative humidity increases, more pores in the porous film are occupied, especially near the surface; then, the diffusion pathway becomes narrower and its resistance increases; as a result, the diffusivity decreases. This problem would be more serious if the connectivity among pores became weaker, or if there were more hydrophilic groups in the film. There may be a stronger connectivity among pores, or fewer hydrophilic groups, in PI film with 23% PI acid concentration, which had the most gradual diffusivity curve.

### 4.3. The Environment Temperature Effect

**Figure 9:**
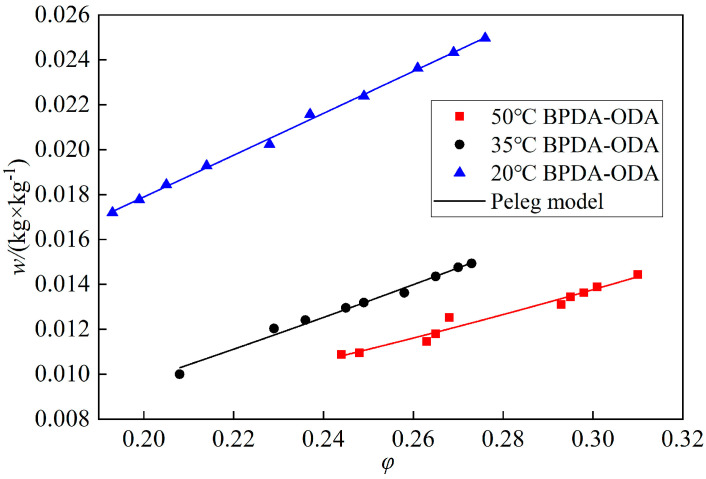
Absorption isothermal curves of BO film in different temperature environments.

In order to investigate the temperature influence on the humidity-sensitive properties of PI films, BO film with an area of 0.01 m^2^ and thickness of 0.025 m was prepared and tested at 20 °C, 35 °C and 50 °C, respectively. Figure 9 shows the results and their w(φ) curves coupled by the Peleg model. As the vapor molecules were charged with more energy and more activity in the higher temperature environments, they could easily diffuse into the deeper pores in the film. The moisture absorption improved with increasing temperature. The average moisture absorption was 2.108 × 10^−2^ kg/kg in the test range corresponding to 50 °C. According to the principle of Le Chatelier and the law of Van’t Hoff [22], the vapor condensation was not obvious in the absorption process due to low relative humidity.

(16) $$w{\left(\varphi \right)}_{20\%\mathrm{BO}}^{35{}^{\circ}C}=5.38275\times 10^{-17}+0.08928\varphi^{1.37605}\;$$

(17) $$w{\left(\varphi \right)}_{20\%\mathrm{BO}}^{50{}^{\circ}C}=3.77133\times 10^{-17}+0.0949\varphi^{1.03658}$$

**Figure 10:**
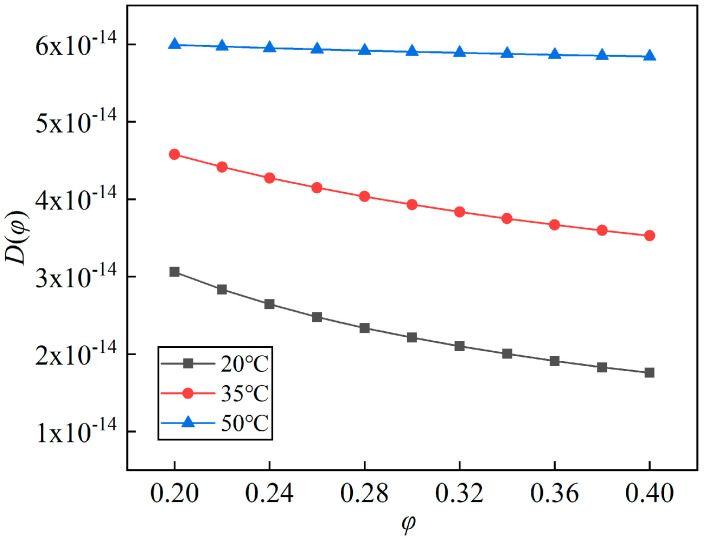
Effective diffusion coefficients of BO film in different environment temperatures.

The coupled formulas of moisture absorption were obtained as Equations (11), (16) and (17), whose correlation coefficients were 0.9697, 0.9873 and 0.9979, respectively. The effective diffusion coefficients were calculated and shown in Figure 10. The environment temperature had a prominent effect on the effective diffusion coefficient of PI films. The higher the temperature, the larger the effective diffusion coefficient. The diffusivity curve at 50 °C was more gentle. The reason could be the same as stated above. The average diffusivities in the range of 20% RH–40% RH were 5.916 × 10^−14^ m^2^/s, 4.051 × 10^−14^ m^2^/s and 2.407 × 10^−14^ m^2^/s, respectively, corresponding to 50 °C, 35 °C and 20 °C.

## 5. Conclusions

The effective diffusion coefficient of moisture sensitive film is valuable for a capacitive humidity sensor, especially for predicting and improving its dynamic characteristics. This paper provides a test system and method by which the test works on several PI films and their analyses were carried out. Conclusions are drawn as follows:

(1) The test system and method is simple and practical, which is helpful to fill in the effective diffusion coefficient database of the porous membranes;
(2) According to the good linearity of the curves in the test range, obvious condensation and hydrophilic groups in the films were not found. Among the four films tested, BB film was the better choice for use as a moisture sensitive film of a capacitive humidity sensor;
(3) On the humidity-sensitive property of PI film, the concentration effect could be indirect, and high temperature had a large impact in the low humidity environment;
(4) The morphology of the film is correlated with the sensing performance, which will be the next research project of our team.

## Figures and Tables

**Table 1:** Main equipment information.

Name	Model	Brand	Quantity
Electronic scales	HZ-104/55S	The United States Huszhi	1
Oil-less air compressor	SQ12	Qiangsheng	1
Temperature and humidity sensor	RS-WS-NO1-SMG-7	Renke	3
Constant temperature humidity chamber	HWHS-225-0	Aodema	1
Barometer	Y-100	Hongqi	3
Voltage-stabilized source	MP6010D	Maisheng	1

**Table 2:** Reagents information.

Reagent	Specification	Manufacturer
BAPP	98%	Shanghai Aladdin Biochemical Technology Co., Ltd., Shanghai, China
PMDA	98%	Shanghai Aladdin Biochemical Technology Co., Ltd., Shanghai, China
ODA	98%	Shanghai Aladdin Biochemical Technology Co., Ltd., Shanghai, China
BPDA	97%	Shanghai Aladdin Biochemical Technology Co., Ltd., Shanghai, China
DMAC	Analytically pure	Shanghai Zhongqin Chemical Reagent Co., Ltd., Shanghai, China

**Table 3:** The characteristic peaks of PI FTIR spectra.

Group	Spectral Characteristic Peak (cm^−1^)
C=O asymmetrical stretching	1779.45, 1775.01, 1776.83
C=O symmetric stretching	1727.01, 1719.67, 1725.32
C-N extension	1388.47, 1375.00, 1378.88
C=O bend	725.66, 738.00, 725.44
benzene ring	1504.8, 1502.30, 1502.60

## Data Availability

Not applicable.
